# External validation of hemoglobin and neutrophil levels as predictors of the effectiveness of ipilimumab plus nivolumab for treating renal cell carcinoma

**DOI:** 10.3389/fonc.2024.1400041

**Published:** 2024-09-02

**Authors:** Shuzo Hamamoto, Yoshihiko Tasaki, Shimpei Yamashita, Junya Furukawa, Kazutoshi Fujita, Ryotaro Tomida, Makito Miyake, Noriyuki Ito, Hideto Iwamoto, Yosuke Sugiyama, Kazumi Taguchi, Takahiro Yasui

**Affiliations:** ^1^ Department of Nephro-urology, Nagoya City University Graduate School of Medical Sciences, Nagoya, Japan; ^2^ Department of Clinical Pharmaceutics, Nagoya City University Graduate School of Medical Sciences, Nagoya, Japan; ^3^ Department of Urology, Wakayama Medical University, Wakayama, Japan; ^4^ Department of Urology, Kobe University Graduate School of Medicine, Kobe, Japan; ^5^ Department of Urology, Kindai University Faculty of Medicine, Osakasayama, Japan; ^6^ Department of Urology, Tokushima University Graduate School of Biomedical Sciences, Tokushima, Japan; ^7^ Department of Urology, Nara Medical University, Kashihara, Japan; ^8^ Department of Urology, Japanese Red Cross Wakayama Medical Center, Wakayama, Japan; ^9^ Division of Urology, Department of Surgery, Tottori University Faculty of Medicine Graduate School of Medicine, Tottori, Japan

**Keywords:** hemoglobin, neutrophil, ipilimumab plus nivolumab, renal cell carcinoma, external validation

## Abstract

**Introduction:**

Pretreatment hemoglobin and neutrophil levels were previously reported to be important indicators for predicting the effectiveness of ipilimumab plus nivolumab (IPI + NIVO) therapy for renal cell carcinoma (RCC). Therefore, we aimed to validate this in a large external cohort.

**Methods:**

In total, 172 patients with RCC who underwent IPI + NIVO treatment at a multicenter setting were divided into three groups according to their pretreatment hemoglobin and neutrophil levels (group 1: non-anemia; group 2: anemia and low-neutrophil; and group 3: anemia and high-neutrophil).

**Results:**

Group 1 showed better survival than groups 2 and 3 (overall survival: 52.3 vs. 21.4 vs. 9.4 months, respectively; progression-free survival: 12.1 vs. 7.0 vs. 3.4 months, respectively).

**Discussion:**

In this large cohort, we validated our earlier observation that hemoglobin and neutrophil levels can be reliable predictors of the effectiveness of IPI + NIVO in advanced RCC. Thus, our approach may aid in selecting the optimal first-line therapy for RCC.

## 1 Introduction

Immune checkpoint inhibitor (ICI) combination therapies and ICI plus vascular endothelial growth factor (VEGF)–targeted therapy are recommended as standard primary treatments for advanced renal cell carcinoma (RCC) by the National Comprehensive Cancer Network guidelines (1–5). Among these therapies, ipilimumab plus nivolumab (IPI + NIVO) is one of the essential treatments for intermediate and patients with poor-risk RCC, as classified by the International Metastatic Renal Cell Carcinoma Database Consortium (IMDC). From a long-term analysis (60 months) in a large clinical trial, the median overall survival (mOS) and median progression-free survival (mPFS) were demonstrated to be 55.7 and 12.3 months, respectively (1).

In RCC, eligibility for treatment selection is determined using the IMDC risk classification (6). In practice, IPI + NIVO has been approved in Japan as the primary treatment for advanced RCC classified as intermediate or poor-risk. Although the IMDC risk classification was reported in the era of VEGF-targeted monotherapy, it may be a valuable predictive marker for ICI therapy, as previous studies have reported that it reflects the clinical outcomes of IPI + NIVO therapy (7). In addition, studies on biomarkers for predicting clinical outcomes are actively being conducted (8, 9). However, validated biomarkers have yet to be identified in the real world.

Our previous report showed that the IMDC risk classification plays a vital role in predicting the effectiveness of IPI + NIVO treatment. Furthermore, we identified hemoglobin and neutrophil levels as the most crucial factors in predicting the clinical outcome of IMDC risk classification (7). However, because our previous study had a limited number of participants and a short observation period, we investigated this observation using a larger external cohort.

## 2 Methods

### 2.1 Study design and treatment

This multicenter retrospective research study included 172 patients who underwent IPI + NIVO (ipilimumab at 1 mg/kg and nivolumab at 240 mg/kg every 3 weeks) treatment between October 2015 and February 2023. All patients were followed up until death or loss of contact. We examined hemoglobin and neutrophil levels after blood sampling before the first course of ICI treatment. OS was defined as the period from treatment to death or the last follow-up. Response Evaluation Criteria in Solid Tumors, version 1.1., was used to evaluate the treatment response to IPI + NIVO therapy. Immune-related adverse events (irAEs) were defined as symptoms suspected of resulting from immune dysregulation based on blood sampling and clinical assessment. IrAEs were graded according to the National Cancer Institute Common Terminology Criteria for Adverse Events, version 5.0.

### 2.2 Statistical analyses

Statistical analyses were performed using EZR for R software (10). Fisher’s exact test was used to calculate the categorial valuables in patient characteristics. Overall survival (OS) and progression-free survival (PFS) were statistically compared using the Kaplan–Meier and log-rank tests. Univariate and multivariate Cox regression analyses were applied to investigate the critical factors for OS and PFS. The *P*-value for statistical significance was set at < 0.05.

## 3 Results

### 3.1 Patient characteristics

**Supplementary Table 1** summarizes the patient characteristics. Patients were divided into three groups according to the pretreatment hemoglobin and neutrophil levels: non-anemia group (group 1: hemoglobin ≥ 12 g/dL; n = 87; 50.6%), anemia and low-neutrophil group (group 2: hemoglobin < 12 g/dL and neutrophil count ≤ 7,000/µL; n = 67; 39.0%), and anemia and high-neutrophil group (group 3: hemoglobin < 12 g/dL and neutrophil count > 7,000/µL; n = 18; 10.4%). The proportions of age, histological subtype, bone metastasis, liver metastasis, lung metastasis, number of courses, response to IPI + NIVO, ≥ grade 3 of irAEs, and patients who discontinued due to irAEs did not differ among the three groups. Patient characteristics, such as sex, IMDC risk group, sarcomatoid change, other metastatic sites, and any grade of irAEs, were significantly different among the three groups. The median follow-up period was 19.9 months (range: 0.5–85.7).

### 3.2 Survival outcomes

**Figure 1:**
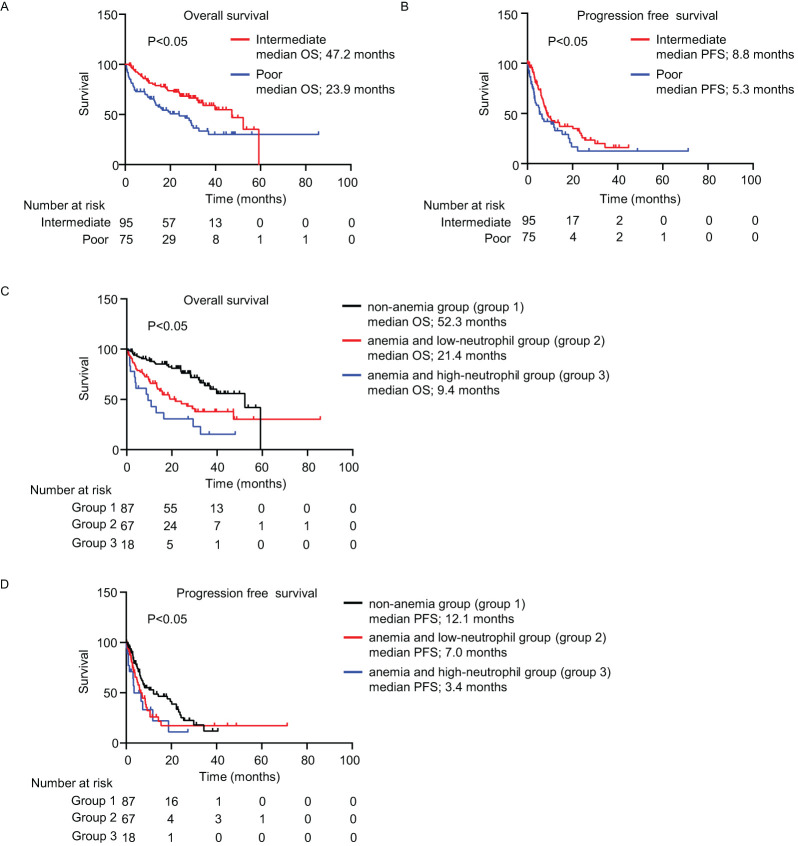
Survival outcomes. **(A–D)** Kaplan–Meier survival curves for **(A)** overall survival (intermediate-risk: n = 95; poor-risk: n = 75); **(B)** progression-free survival (intermediate-risk: n = 95; poor-risk: n = 75); **(C)** overall survival (non-anemia (group 1): n = 87; anemia and low-neutrophil (group 2): n = 67; anemia and high-neutrophil (group 3): n = 18; and **(D)** progression-free survival among three groups. **(A–D)** Log-rank test. IMDC, International Metastatic Renal Cell Carcinoma Database Consortium; OS, overall survival; PFS, progression-free survival.

The mOS and mPFS for patients with intermediate-risk were significantly longer than those with poor-risk (OS: 47.2 vs. 23.9 months; PFS: 8.8 vs. 5.3 months; *P* < 0.05; **Figures 1A, B**). The mOS in group 1 was significantly longer than in groups 2 and 3 (52.3 vs. 21.4 vs. 9.4 months, respectively; *P* < 0.05; **Figure 1C**). Similar to the OS result, the mPFS in group 1 was significantly longer than that in groups 2 and 3 (12.1 vs. 7.0 vs. 3.4 months, respectively; *P* < 0.05; **Figure 1D**). The absence of anemia was an independent factor that lowered the risk of disease progression in the univariate [*P* < 0.05; hazard ratio (HR), 0.62; 95% confidence interval (CI), 0.41–0.95] and multivariate (*P* < 0.05; HR, 0.60; 95% CI, 0.37–0.97) Cox regression analyses (**Table 1**). Consistently, the absence of anemia was a factor for indicating improved OS in both the univariate (*P* < 0.05; HR, 0.38; 95% CI, 0.23–0.61) and multivariate (*P* < 0.05; HR, 0.31; 95% CI, 0.17–0.56) Cox regression analyses (**Supplementary Table 2**). Although the univariate Cox regression analyses did not show that anemia and a low neutrophil count increased the risk for disease progression (*P =* 0.19; HR, 1.32; 95% CI, 0.86–2.03), anemia and a high neutrophil count did tend to increase the risk for disease progression (*P =* 0.09; HR, 1.67; 95% CI, 0.91–3.08; **Table 1**).

**Table 1:** Univariate and multivariate Cox regression analysis of factors predicting progression free survival.

	Univariate			Multivariate		
	HR	95% CI	*P*-value	HR	95% CI	*P-*value
Age: ≥65 years	0.98	0.64–1.51	0.94	0.92	0.57–1.47	0.74
Sex: male	0.72	0.43–1.19	0.20	0.53	0.29–0.99	<0.05
Diagnosis-to-treatment time<1 year	1.19	0.69–2.06	0.51	0.94	0.50–1.76	0.85
KPS: ≥80	0.60	0.37–0.97	<0.05	1.35	0.77–2.36	0.28
Calcium: >upper limit of normal	1.03	0.49–2.14	0.92	0.60	0.24–1.52	0.28
Platelets: >upper limit of normal	1.31	0.81–2.12	0.26	1.46	0.83–2.58	0.18
Histology: clear	0.64	0.41–0.98	<0.05	0.81	0.48–1.36	0.44
Sarcomatoid change: yes	0.96	0.52–1.78	0.91	1.50	1.07–2.12	<0.05
Metastasis site, liver: yes	1.45	0.84–2.49	0.17	1.73	0.91–3.28	0.09
Metastasis site, lung: yes	1.11	0.73–1.69	0.61	1.20	0.73–1.95	0.45
Metastasis site, bone: yes	1.08	0.69–1.69	0.71	1.32	0.81–2.16	0.25
Metastasis site, others: yes	1.21	0.78–1.86	0.37	1.05	0.63–1.76	0.82
Non-anemia group: yes	0.62	0.41–0.95	<0.05	0.60	0.37–0.97	<0.05
Anemia and low-neutrophil group: yes	1.32	0.86–2.03	0.19			
Anemia and high-neutrophil group: yes	1.67	0.91–3.08	0.09			

## 4 Discussion

In this study, we validated the pretreatment hemoglobin and neutrophil levels as biomarkers to analyze the clinical effectiveness of IPI + NIVO therapy for RCC. We used an external cohort with more participants, and the observation period was longer than that of our previous study (7). Consistent with our previous findings, the mOS and mPFS were best for group 1 and worst for group 3 (**Figures 1C, D**, **Table 1**; **Supplementary Table 2**). Moreover, group 1 had a lower risk of disease progression and poorer survival than the other factors of IMDC classification, as per the multivariate Cox regression analysis results (**Table 1**; **Supplementary Table 2**).

A large European cancer anemia survey found that approximately 70% of patients with cancer had anemia (11). There are various factors that cause anemia, such as tumor extension into bone marrow, chemotherapy, and deficiency of iron, vitamin, and erythropoietin (12). As anemia causes poor performance status, quality of life, and prognosis, it is a crucial prognostic indicator for various cancers (11, 13, 14). Several reports have also demonstrated that anemia is related to poor prognosis in patients with RCC. For example, hemoglobin levels were closely related to survival in patients who received tyrosine kinase inhibitors (15). Furthermore, 41.6% of patients who registered in the CheckMate 214 trial had anemia before treatment, and a univariate analysis showed that anemia significantly increased the risk of poor OS (16). Consistent with our previous studies (7, 15, 16), 49.4% of patients in the current study had anemia before treatment (**Supplementary Table 1**), and group 1 was associated with better OS and PFS (**Figures 1C, D**, **Table 1**; **Supplementary Table 2**). The results of the current study, taken together with previous studies, suggest that anemia is a crucial factor for predicting survival.

Neutrophil levels are also associated with RCC prognosis. Patients with RCC with a neutrophil-to-lymphocyte ratio (NLR) cutoff of > 4.0 who received an ICI had a 1.65-fold increased risk of poor OS (17). In addition, the NLR was correlated with poor OS in patients with RCC who underwent nephrectomy (18). One reason for the association between neutrophil levels and prognosis is that neutrophils promote tumor progression (19). Neutrophils infiltrated into tumor by chemokines that are secreted by tumor cells, immune cells, and cancer-associated fibroblasts (19). Tumor-infiltrating neutrophils secrete cytokines, chemokines, reactive oxygen species, and oncostatin M in tumor, and these factors promote tumor progression, angiogenesis, and metastasis (19). Moreover, as shown in a previous study, factors that are secreted by tumor-infiltrating neutrophils are regulated by DNA demethylation and super-enhancer formation (20).

The five treatments with ICI combination therapy including ipilimumab plus nivolumab are recommended as a standard first-line therapy for RCC according to the National Comprehensive Cancer Network guidelines (1–5). However, it difficult for physician to determine the optimal treatment for each patient among five treatments. There are currently a number of studies on biomarkers to evaluate the effectiveness and prognosis of ICI combination therapy, such as genetic expression/mutation, blood parameters, and adverse effects; there is still no innovative biomarker to determine the optimal treatment (9, 21–25). Among those biomarkers, the IMDC risk classification is a useful biomarker to evaluate the effectiveness and prognosis of ipilimumab plus nivolumab therapy, and our data support this (7). On the other hand, IMDC risk classification was created in the VEGF-targeted monotherapy and needs to be more refined for the ipilimumab plus nivolumab therapy. In the current study, the mOS and mPFS of group 1 with intermediate IMDC risk was significantly longer than that of the groups 2 and 3 (mOS: 52.3 vs. 18.8 vs. 9.4 months; mPFS: 14.2 vs. 7.2 vs. 3.2 months, respectively; *P* < 0.05; **Supplementary Figure 1**). The mOS and mPFS of patients with poor IMDC risk was 28.3 and 5.3 months in group 1, 26.5 and 5.2 months in group 2, and 10.8 and 6.5 months in group 3 (mOS: *P* = 0.07; mPFS: *P* = 0.75; **Supplementary Figure 2**). These data indicated that hemoglobin and neutrophil can stratify patients who are classified by IMDC risk, especially to intermediate risk, and predict the effectiveness and prognosis of ipilimumab plus nivolumab therapy. Additionally, Takemura et al. discussed the possibility that platelets and calcium may not be important IMDC risk classification in a different way than us (26). Although the patients enrolled in their study (26) had different patient backgrounds from our study because of the inclusion of ICI plus VEGF–targeted combination therapy, their study supports our results and considerations. In brief, hemoglobin and neutrophil may be rational biomarker to evaluate the effectiveness and prognosis of ipilimumab plus nivolumab therapy.

The mOS and mPFS in group 1 were similar to those in the CheckMate 214 trial (1). In contrast, the mOS and mPFS in group 3 were significantly worse than in that trial. These data suggest that IPI + NIVO therapy should be recommended for patients without anemia; however, patients with anemia and high neutrophil counts might be considered for combinations of ICI and VEGF-targeted therapy. In patients with anemia and low neutrophil counts, physicians should consider treatment options based on performance status and disease progression.

This study had a methodological limitation. Specifically, we could not control for bias in patient selection because this was a retrospective study. Thus, we plan to confirm our findings using a prospective study.

In conclusion, we report robust results demonstrating that hemoglobin and neutrophil values before treatment are important predictors of the effectiveness of IPI + NIVO therapy in patients with RCC.

## Data Availability

The raw data supporting the conclusions of this article will be made available by the authors, without undue reservation.
