# Supplementary material for: External validation of hemoglobin and neutrophil levels as predictors of the effectiveness of ipilimumab plus nivolumab for treating renal cell carcinoma
Source: Front Oncol. 2024 Sep 2;14:1400041. doi: 10.3389/fonc.2024.1400041 (PMC11402603; doi:10.3389/fonc.2024.1400041)
Supplement: Supplementary file 1 [file Table1.docx]

**Supplementally Table 1. Clinical features of patients**

| Characteristics | Non-anemia  group | anemia+Neutro-Low group | anemia+Neutro-High group | *P* value |
| --- | --- | --- | --- | --- |
| Total, n (%) | 87 (50.6) | 67 (39.0) | 18 (10.4) |  |
| Age, n (%) |  |  |  | 0.05 |
| <65 years | 35 (40.2) | 15 (22.4) | 7 (38.9) |  |
| ≥65 years | 49 (56.3) | 50 (74.6) | 11 (61.1) |  |
| Sex, n (%) |  |  |  | <0.05 |
| Male | 75 (86.2) | 47 (70.1) | 16 (88.9) |  |
| Female | 12 (13.8) | 20 (29.9) | 2 (11.1) |  |
| IMDC risk group, n (%) |  |  |  | <0.05 |
| Intermediate | 68 (78.2) | 26 (38.8) | 1 (5.6) |  |
| Poor | 17 (19.5) | 41 (61.2) | 17 (94.4) |  |
| Histological subtype, n (%) |  |  |  | 0.13 |
| Clear cell | 60 (69.0) | 46 (68.7) | 8 (44.4) |  |
| Non-clear cell | 23 (26.4) | 17 (25.4) | 9 (50.0) |  |
| Papillary | 9 | 6 | 3 |  |
| Chromophobe | 4 | 0 | 1 |  |
| Bellini duct carcinoma | 1 | 0 | 0 |  |
| Spindle | 2 | 0 | 0 |  |
| Others | 7 | 11 | 5 |  |
| Unknown | 4 (4.6) | 4 (5.9) | 1 (5.6) |  |
| Sarcomatoid change |  |  |  | <0.05 |
| No | 80 (92.0) | 59 (88.1) | 14 (77.8) |  |
| Yes | 7 (8.0) | 8 (11.9) | 4 (22.2) |  |
| Metastasis site, Bone |  |  |  | 0.51 |
| No | 60 (69.0) | 46 (68.7) | 10 (55.6) |  |
| Yes | 27 (31.0) | 21 (31.3) | 8 (44.4) |  |
| Metastasis site, liver, n (%) |  |  |  | 0.86 |
| No | 73 (83.9) | 58 (86.6) | 16 (88.9) |  |
| Yes | 14 (16.1) | 9 (13.4) | 2 (11.1) |  |
| Metastasis site, lung, n (%) |  |  |  | 0.26 |
| No | 36 (41.4) | 29 (43.3) | 4 (22.2) |  |
| Yes | 51 (58.6) | 38 (56.7) | 14 (77.8) |  |
| Metastasis site, others, n (%) |  |  |  | <0.05 |
| No | 38 (43.7) | 29 (43.3) | 3 (16.7) |  |
| Yes | 49 (56.3) | 38 (56.7) | 15 (83.3) |  |
| Number of courses |  |  |  | 0.63 |
| 1 | 7 (8.0) | 10 (14.9) | 4 (22.2) |  |
| 2 | 14 (16.1) | 10 (14.9) | 2 (11.1) |  |
| 3 | 14 (16.1) | 9 (13.4) | 4 (22.2) |  |
| 4 | 52 (59.8) | 38 (56.7) | 8 (44.4) |  |
| Response to ipilimumab plus nivolumab, n (%) |  |  |  | 0.48 |
| Complete response | 10 (11.5) | 6 (9.0) | 1 (5.6) |  |
| Partial response | 29 (33.3) | 24 (35.8) | 6 (33.3) |  |
| Stable disease | 27 (31.0) | 16 (23.9) | 2 (11.1) |  |
| Progressive disease | 16 (18.4) | 18 (26.9) | 7 (38.9) |  |
| Not evaluable | 5 (5.7) | 3 (4.4) | 2 (11.1) |  |
| Incidence of any grade of irAEs, n (%) |  |  |  | <0.05 |
| No | 19 (21.8) | 28 (41.8) | 10 (55.6) |  |
| Yes | 68 (78.2) | 39 (58.2) | 8 (44.4) |  |
| Incidence of ≥grade3 of irAEs, n (%) |  |  |  | 0.06 |
| No | 48 (55.2) | 48 (71.6) | 13 (72.2) |  |
| Yes | 39 (44.8) | 19 (28.4) | 5 (27.8) |  |
| Number of patients who discontinued due to irAEs, n (%) |  |  |  | 0.11 |
| No | 68 (78.2) | 52 (77.6) | 10 (55.6) |  |
| Yes | 19 (21.8) | 15 (22.4) | 8 (44.4) |  |

IMDC: International Metastatic Renal Cell Carcinoma Database Consortium; irAEs: immune-related adverse events

**Supplementally Table 2. Univariate and multivariate cox regression analysis of factors predicting overall survival**

|  | Univariate | | | Multivariate | | |
| --- | --- | --- | --- | --- | --- | --- |
|  | HR | 95%CI | *P* value | HR | 95%CI | *P* value |
| Age: ≥65 years | 0.98 | 0.61-1.57 | 0.93 | 0.71 | 0.41-1.23 | 0.23 |
| Sex: male | 0.77 | 0.45-1.31 | 0.34 | 0.67 | 0.35-1.25 | 0.21 |
| Diagnosis-to-treatment time<1 year | 1.35 | 0.72-2.62 | 0.33 | 1.14 | 0.55-2.34 | 0.71 |
| KPS: ≥80 | 0.48 | 0.29-0.77 | <0.05 | 1.31 | 0.72-2.38 | 0.36 |
| Calcium: >upper limit of normal | 0.90 | 0.43-1.88 | 0.78 | 0.42 | 0.16-1.10 | 0.07 |
| Platelets: >upper limit of normal | 1.38 | 0.82-2.31 | 0.21 | 0.88 | 0.45-1.72 | 0.71 |
| Histology: clear | 0.40 | 0.25-0.63 | <0.05 | 0.43 | 0.24-0.78 | <0.05 |
| Sarcomatoid change: yes | 1.46 | 0.75-2.85 | 0.26 | 1.20 | 0.83-1.73 | 0.31 |
| Metastasis site, liver: yes | 1.51 | 0.87-2.64 | 0.13 | 1.54 | 0.81-2.91 | 0.18 |
| Metastasis site, lung: yes | 1.56 | 0.97-2.53 | 0.06 | 1.95 | 1.13-3.38 | <0.05 |
| Metastasis site, Bone: yes | 0.99 | 0.61-1.58 | 0.96 | 0.90 | 0.50-1.62 | 0.74 |
| Metastasis site, others: yes | 1.39 | 0.88-2.20 | 0.15 | 1.24 | 0.72-2.13 | 0.42 |
| Non-anemia group: yes | 0.38 | 0.23-0.61 | <0.05 | 0.31 | 0.17-0.56 | <0.05 |
| Anemia and low-neutrophil group: yes | 1.68 | 1.07-2.64 | <0.05 |  |  |  |
| Anemia and high-neutrophil group: yes | 2.79 | 1.56-5.00 | <0.05 |  |  |  |

HR: hazard ratio; 95%CI: 95% confidence interval; KPS: Karnofsky Performance Status
