# Supplementary figures and images for: External validation of hemoglobin and neutrophil levels as predictors of the effectiveness of ipilimumab plus nivolumab for treating renal cell carcinoma
Source: Front Oncol. 2024 Sep 2;14:1400041. doi: 10.3389/fonc.2024.1400041 (PMC11402603; doi:10.3389/fonc.2024.1400041)

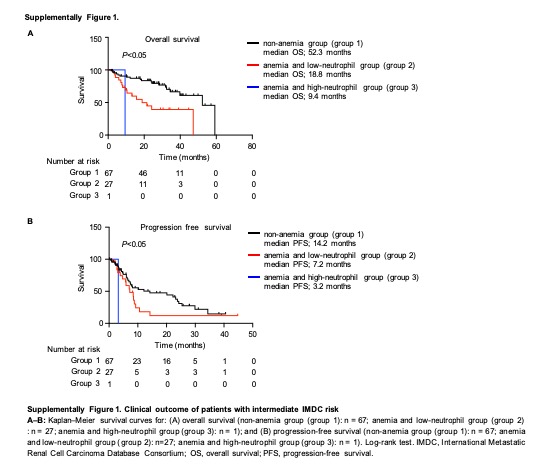

Supplement: Supplementary file 2 [file Image1.jpeg]

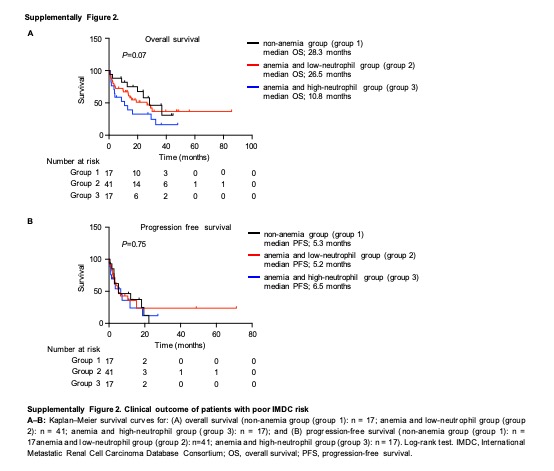

Supplement: Supplementary file 3 [file Image2.jpeg]
